# Involvement in Decision-Making for Daily Care and Cognitive Decline among Older Adults Who Need Care in Japan

**DOI:** 10.1093/geroni/igab046.2628

**Published:** 2021-12-17

**Authors:** Ayane Komatsu, Takeshi Nakagawa, Taiji Noguchi, Tami Saito

**Affiliations:** 1 Center for Gerontology and Social Science, Research Institute, National Center for Geriatrics and Gerontology, Obu, Aichi, Japan; 2 National Center for Geriatrics and Gerontology, Obu, Aichi, Japan

## Abstract

Effective decision-making regarding daily care for older adults with needs could reduce the risk of dementia by preventing loss of motivation and improving care quality. However, empirical studies are scarce, particularly in non-Western countries with different socio-cultural backgrounds. By using 2-year longitudinal data of older Japanese adults aged 65 years and above who were receiving care at home, as well as of their family caregivers, we examined the association of involvement in decision-making with the onset of cognitive decline among older Japanese adults requiring care. The analysis included 219 cases of individuals with normal cognition and no missing variables at baseline and responded to the follow-up survey. An MMSE score of 23 or lower at follow-up was defined as the onset of cognitive decline. The level of involvement in decision-making was assessed by one item and dichotomized (not involved/involved). The covariates were age, gender, education, MMSE score, eligibility level for long-term care, and others at baseline. At baseline, 67.1% were 75 years or older, 58.9% were female, and 91.8% responded being “involved” in the decision-making. The incidence of cognitive decline at follow-up was 30.6%. The multivariable logistic regression analysis showed that involvement in decision-making (OR=0.298 [95% CI: 0.10-0.88], p=0.029) was negatively and significantly associated with the onset of cognitive decline. Our findings show the importance of involvement in the decision-making for daily care to reduce the risk of subsequent cognitive decline in older adults requiring care, even in a culture of familism.

